# Compensatory mechanisms in response to induced hypothyroidism in the late gestation pig fetus^†^

**DOI:** 10.1093/biolre/ioad024

**Published:** 2023-02-22

**Authors:** Erin K Ison, Coral E Kent-Dennis, James Fazioli, Margaret K Mulligan, Audrey Pham, J Alex Pasternak

**Affiliations:** Department of Animal Science, Purdue University, West Lafayette, IN, USA; Postdoctoral fellow, Oak Ridge Institute for Science and Education; Department of Animal Science, Purdue University, West Lafayette, IN, USA; Department of Animal Science, Purdue University, West Lafayette, IN, USA; Department of Animal Science, Purdue University, West Lafayette, IN, USA; Department of Animal Science, Purdue University, West Lafayette, IN, USA

**Keywords:** methimazole, hypothyroidism, maternal, fetal, metabolism

## Abstract

To understand the effect of fetal thyroid gland disruption on development in swine, we evaluated thyroid hormone levels, growth and developmental characteristics, and gene expression associated with thyroid hormone metabolism in late gestation fetuses exposed to methimazole (MMI). Pregnant gilts were given either oral MMI or equivalent sham from gestation day 85–106 (*n* = 4/group), followed by intensive phenotyping of all fetuses (*n* = 120). Samples of liver (LVR), kidney (KID), fetal placenta (PLC), and the corresponding maternal endometrium (END) were collected from a subset of fetuses (*n* = 32). Fetuses exposed to MMI *in utero* were confirmed hypothyroid, with a significant increase in thyroid gland size, goitrous thyroid histology, and dramatically suppressed thyroid hormone in serum. In dams, no differences in temporal measurements of average daily gain, thyroid hormone, or rectal temperatures relative to controls suggests that MMI had little effect on maternal physiology. However, fetuses from MMI-treated gilts exhibited significant increases in body mass, girth, and vital organ weights, but no differences in crown-rump length or bone measurements suggesting non-allometric growth. The PLC and END showed a compensatory decrease in expression of inactivating deiodinase (DIO3). Similar compensatory gene expression was observed in fetal KID and LVR with a downregulation of all deiodinases (DIO1, DIO2, DIO3). Minor alterations in the expression of thyroid hormone transporters (SLC16A2 and SLC16A10) were observed in PLC, KID, and LVR. Collectively, MMI crosses the PLC of the late gestation pig, resulting in congenital hypothyroidism, alterations in fetal growth, and compensatory responses within the maternal fetal interface.

## Teaser

Methimazole crosses the late gestation pig placenta resulting in congenital hypothyroidism, non-allometric fetal growth, and compensatory changes in thyroid hormone metabolites.

## Graphical abstract

**Figure:**
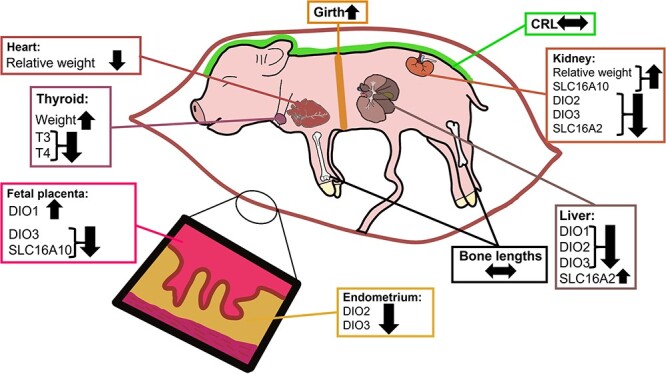

## Introduction

The rate of growth and development in fetal swine increases exponentially during late gestation [1]. This increased growth rate is driven by fetal upregulation of various endocrine signals, including insulin-like growth factor 1 [2], growth hormone [3], and thyroid hormone [4]. To accommodate this rapid growth and the associated nutrient requirements, the placenta must be adaptable in both structure [5] and function [6]. Early in gestation, the porcine placenta is comprised of simple interdigitations of intact maternal and fetal tissues creating a layered structure that is relatively restrictive to the transport of nutrients and large molecules [7]. In response to increasing fetal demands, the placenta begins to fold, increasing in complexity and surface area throughout gestation, which increases nutrient transport [5, 8]. Additional adaptation occurs at the molecular level, with an upregulation in angiogenic signaling in response to both gestational progression [9] and pathogenic challenge [10]. Similarly, placental epithelial barrier function is altered by infection [11] and in response to the differential growth rate [12]. However, the capacity of the porcine placenta to compensate for disruption in fetal endocrinology has not been demonstrated [13].

Thyroid hormone is equally critical for both terminal maturation and accretion of mass and is upregulated during late gestation in the pig fetus [4, 14]. This hormone group shares a common two-ring structure, with various forms distinguished by the presence and location of 2–4 iodine molecules. The most abundant of which, Thyroxine (T4), is produced in the thyroid gland along with traces of its more bioactive metabolite triiodothyronine (T3). Along with T3, other derivatives of T4 are produced in extra-thyroidal tissues, including the largely inactive reverse T3 (rT3) and the diiodothyronines (T2), 3,3 T2, 3,3’T2, and 3,5 T2. The bioactive forms of thyroid hormone are most well-known for regulating adult metabolism [15]. However, they are also necessary for normal fetal growth and organ development [16]. A deficiency of fetal thyroid hormone during gestation has long been known to cause cretinism in humans [17] or similar congenital defects in swine [18], cattle [19], and sheep [20]. Congenital hypothyroidism is also linked to cognitive disorders in rats and humans [21]. The fetuses of some species, such as humans and rats, rely heavily on the dam to supply bioactive thyroid hormone until the fetal thyroid gland develops and becomes autonomous in mid-gestation [22]. There is evidence that species such as the pig are less dependent on maternal thyroid hormone supply, as the fetal thyroid gland in the develops and becomes autonomous during early gestation [4]. Furthermore, the pig placenta has an enzymatic barrier to maternal thyroid hormone, which deactivates the hormone from the dam, limiting stimulation in the fetus [23].

While thyroid hormone is normally regulated by homeostatic mechanisms of the hypothalamic-pituitary-thyroid axis, stress from trauma, illness, or starvation is known to cause an adaptive allostatic state known as non-thyroidal illness syndrome (NTIS) [24]. NTIS also develops in response to infection with pathogens such as porcine reproductive and respiratory syndrome virus (PRRSV), which impacts the thyroid system of both post-natal and fetal pigs [25, 26]. In addition to regulating thyroid hormones, the production of hormones within the thyroid gland can be affected by goitrogens. Natural goitrogens include glucosinolates found in rapeseed, which are iodine antagonists and disrupt the thyroid system by preventing iodine absorption into thyroid follicular cells, thereby restricting T4 production [27]. Similarly, synthetic goitrogens, or anti-thyroid drugs (ATDs), including propylthiouracil (PTU), and methimazole (MMI), are commonly used in human medicine to treat hyperthyroidism [28]. However, when given to a euthyroid animal, these compounds severely depress normal thyroid hormone production and result in a hypothyroid state [29, 30]. In general, ATDs prevent thyroid hormone production either by restricting iodine availability or through inhibition of iodine incorporation onto T4 precursors. Specifically, MMI and PTU inhibit the enzyme thyroid peroxidase, preventing iodination of the T4 precursor, thyroglobulin within the normally eosinophilic colloid of the thyroid follicle [31]. Both of these drugs are known to cross the hemochorial placenta of humans and rats during pregnancy [32, 33]. However, there is some ambiguity regarding optimal ATD for use during pregnancy, due to the potential for congenital defects and long-term negative effects on the fetus [34, 35].

To understand the effect of temporary disruption of the fetal thyroid gland on thyroid hormone levels and development, we used maternal, oral administration of MMI to induce hypothyroidism in the fetal pig. First, we evaluated the impact of MMI-induced hypothyroidism on pregnant gilts through average daily gain (ADG), temperature regulation, and thyroid status. The resulting litters were assessed for changes in the frequencies of live and viable fetuses. Next, we investigated the impact of MMI on fetal growth and development through body weight, organ weights, crown-rump length (CRL), girth, and long bone lengths. To determine the thyroid status of the fetus, we evaluated the thyroid gland size, histology, and T4 levels in serum. Finally, we investigated the potential of the fetus to compensate for thyroid disruption by evaluating the gene expression of three deiodinases (DIO1, 2, 3) known to metabolize thyroid hormone and two prominent thyroid hormone transporters SLC16A2 and SLC16A10. To evaluate possible alterations in thyroid hormone metabolism or excretion during hypothyroidism, gene expression was measured in the fetal liver (LVR) and kidney (KID), which are two key organs known to process thyroid hormone. Potential compensation of the placental enzymatic barrier was investigated by measuring gene expression in the fetal portion of the placenta (PLC) and the corresponding maternal endometrium (END). Overall, we hypothesize that maternal MMI treatment will induce fetal hypothyroidism, alter developmental phenotype and result in compensatory gene expression within the MFI and fetal organs.

## Materials and methods

### Animals

A total of eight healthy terminal cross gilts (F1 x Durroc) with average body condition were synchronized with 17.6 mg of Altrenogest per day for 17 days (Merck Animal Health, Kenilworth, NJ, USA) and housed at the Animal Sciences Research and Education Center (ASREC) at Purdue University. All animals were confirmed pregnant by transcutaneous ultrasound at days 30 and 80 of gestation and tested for the presence of PRRSV using previously described methods [36]. At day 83 of gestation the average weight of all gilts was 190.8 kg with a range of 164–219 kg. Gilts were randomly assigned into either a control (CON) or treatment (TRT) group (*n* = 4/group) on gestation day 85 (trial day 0). Before daily feedings, gilts of both groups were provided with 200 g of feed sweetened with a pre-defined volume of corn syrup that was either untreated (CON) or supplemented with 5 mg/kg of MMI (Sigma-Aldrich, St. Louis, MO, USA). This does of MMI was chosen base on prior work which indicated 5 mg/kg/day was sufficient to suppress serum T3 and T4 in post-natal swine [37]. Behavior based on activity and appearance based on skin color and coat roughness were noted daily to maintain a record of the gilts’ well-being. Body weights were recorded weekly, treatment doses adjusted accordingly, and skin and rectal temperatures were recorded biweekly between 8:00 and 9:30 a.m. Blood samples were collected on trial days –3 (pre-treatment), 7, and 21. All gilts were humanely euthanized on gestation day 106 by captive bolt, followed by exsanguination. This study was done in compliance with Institutional Animal Care and Use Committee regulations and approved by the Purdue Animal Care and Use Committee (Protocol #2103002122).

### Blood and tissue collection

Immediately following gilt euthanasia, the thyroid was removed and weighed, and the pregnant uterus was carefully extracted, linearized, and dissected starting at the ovarian end of the left and right horns to the cervix, being diligent to preserve the fetal position and umbilical attachments to the tract. All fetuses (*n* = 120) were individually examined, sexed, and classified based on preservation status as reported in Ladinig et al. [36] and Malgarin et al. [38]. In short, fetuses that possessed a pulse within the umbilical cord and appeared physically normal were classified as viable (*n* = 73). Those with mild yellow-brown material on their face or body were classified as meconium-stained (*n* = 40). Fetuses with no umbilical pulse were classified as dead and either mummified (*n* = 5) or autolyzed (*n* = 2). Dead fetuses were accounted for in litter size but excluded from further analysis due to organ and body degradation. The fetuses were numbered sequentially, beginning from the ovary of the left and right uterine horns and moving toward the cervix.

**Figure 1:**
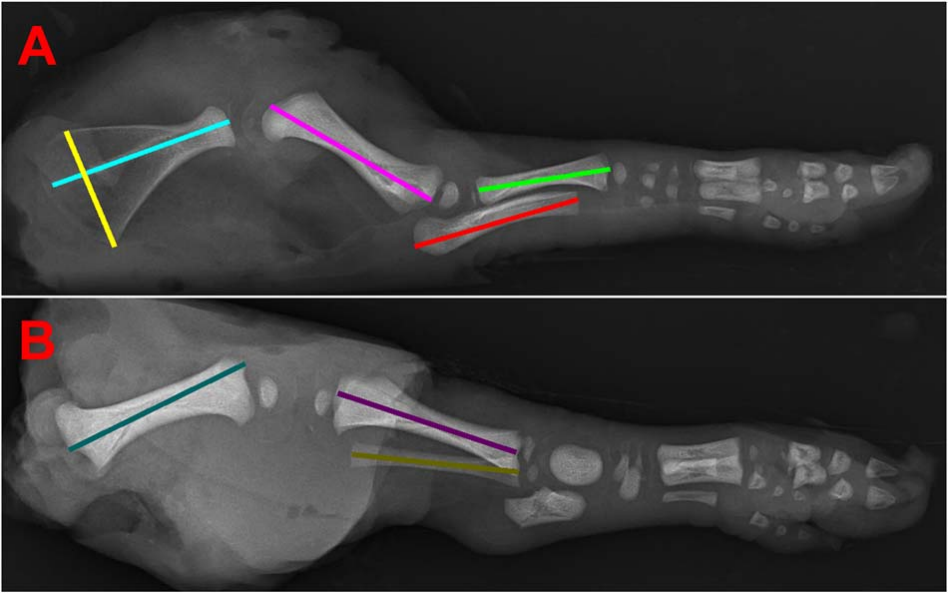
Images of fetal morphometric and forelimb and hindlimb bone measurements. (A) Radiograph of fetal forelimb used to measure width of scapula (yellow), length of scapula (light blue), humerus (pink), ulna (red), and radius (green). Long bones were measured for length by using a straight line through the median record the distance from the proximal ends to the distal ends. Scapula width was measured by a straight line that ran from the tip of the inferior angle to the tip of the superior angle. The length of the scapula was recorded by a straight line dissecting the bone along the central ridge. (B) Radiograph of fetal hindlimb used to measure length of femur (teal), tibia (purple), and fibula (gold).

The most centrally located viable male and female fetuses from each uterine horn were selected as a subset (*n* = 4/gilt) for intensive sampling, and their umbilical attachment to the uterus was marked for subsequent collection. However, three meconium-stained fetuses were chosen because of a lack of viable fetuses within a uterine horn. Blood was collected from the axillary artery of all fetuses immediately after removal from the maternal tract, and serum was later separated and stored at −20°C. Fetal body weights were recorded post-blood collection, and morphometric images and analysis were done similar to that reported in Ko et al. [39]. In short, each fetus was imaged in left and right lateral recumbence using a fixed and level camera, with calibration chips included in each image. Measurements of the CRL and girth were later determined using a semi-automated macro function developed for ImageJ, and the values from left and right lateral recumbencies averaged. The thyroid, cervical thymus, heart, LVR, lungs, KIDs, spleen, and brain from all fetuses were then removed and weighed. The corresponding placenta and endometrium were collected from the select fetuses by placing a section of maternal fetal interface (MFI) on ice until the two layers could be peeled apart. All tissues sampled were packaged individually and snap-frozen in liquid nitrogen, then stored at −80°C until further ribonucleic acid (RNA) analysis, except for thyroid, which was fixed in 10% neutral buffered formalin and sent to the Purdue Histology Research Laboratory for paraffin embedment and staining with hematoxylin and eosin. To assess bone development in select fetuses, the right fetal forelimb and hindlimb were frozen at −20°C until radiographic images were taken. Bones in the forelimb (scapula, humerus, ulna, and radius) and hindlimb (femur, tibia, and fibula) were normalized and measured for length with another semi-automated macro function in ImageJ as shown in Figure 1.

### Thyroid hormone assay

Maternal and fetal total T4 and T3 concentration in serum was determined as previously described in swine [40], using commercially available T4 Enzyme Immunoassay (EIA) Test Kit and T3 EIA Test Kits as described by the manufacturer (MP Biomedicals, Solon, OH, USA). In short, an aliquot of 25 μL per serum sample for T4 and 50 μL for T3 was analyzed in duplicate with two independent pools of swine serum serving as inter-assay controls. Maternal serum was analyzed at three time points (*n* = 8/time point) and a subset of fetal serum was analyzed at trial day 21 (*n* = 56). The inter- and average intra-assay coefficients of variation were 9.23% and 2.07%, respectively, for T4 and 5.62% and 4.40% for T3. The limit of detection was 6.44 nmol/L for T4 and 0.1936 nmol/L for T3.

### RNA extraction and real-time qPCR

Samples of the maternal component of the placenta (END), the fetal component of the PLC, fetal LVR, and fetal KID from the select fetus subset (*n* = 32) were chosen for analysis of gene expression by quantitative polymerase chain reaction (qPCR). The frozen samples were ground under liquid nitrogen using a pre-frozen mortar and pestle. A double precipitation protocol with TRIzol (Thermofisher, Waltham, MA, USA) was used to extract the total RNA from the ground samples. Once extracted, the samples were quantified using a Nanodrop ND-1000 (Thermo Fisher Scientific), and electrophoresis on denaturing agarose gels was used to establish RNA integrity. The Turbo deoxyribonucleic acid (DNA)-free Kit (Invitrogen) and 0.5 μL of RNaseOUT (Thermo Fisher Scientific) were used to DNase treat a 20 μL aliquot of each total RNA sample. The High-Capacity cDNA Reverse Transcription Kit (Applied Biosystems, Foster City, CA, USA) was used to reverse transcribe 2 μg of DNase-treated RNA per sample. Current RefSeq mRNA sequences (Table 1) were referenced to design gene-specific primers covering all predicted transcript variants for genes of interest and housekeeping genes. The BLAST-like alignment tool (BLAT) relative to the *Sus Scrofa* 11.1 assembly was used to identify and design primers to span exon–exon junctions. For each target, primers were determined to have an initial efficiency within the range of 95–105%, based on a five point serial dilution of pooled complimentary DNA (cDNA). The melting curves for each target showed no evidence of multiple amplicon products. Relative abundance PCR was completed using 10 ng equivalent cDNA per reaction in duplicate, including no-template negative control, with SsoAdvanced Universal SYBR Green SuperMix (Bio-Rad) in 96-well plates on a CFX Connect Real-Time System (Bio-Rad).

**Table 1:** Gene-specific primer sequences, including housekeeping genes, used for qPCR.

*Pathway*	*Official symbol*	*Gene ID*	*Forward primer*	*Reverse primer*	*Annealing temp (°C)*	*Amplicon length (bp)*	*Target RefSeq*
*Deiodination*	DIO1	414380	5’-ACTTCATGCAAGGCAACAGG-3’	5’-GGTCCTGGAGATTCTGGTGA-3’	61	213	NM_001001627.1
	DIO2	414379	5’-CTCGGTCATTCTCCTCAAGC-3’	5’-TCACCTGTTTGTAGGCATCG-3’	61	140	NM_001001626.2
	DIO3	414378	5’-CCTATCTGCGTGTCTGACGA-3’	5’-GCCTGCTTGAAGAAATCCAG-3’	61	92	NM_001001625.2
*Transportation*	SLC16A2	100513513	5’-AGTGGAGTTCCAAGCAGCAT-3’	5’-AGCCCAAACGATCAGTGAAT-3’	61	95	XM_021080455.1
	SLC16A10	100513770	5’-CACCCATTGCAGGGTTACTC-3’	5’-TATGGAGCCAAGGGATGAAA-3’	61	117	XM_021091212.1
*Housekeeping*	ACTB	414396	5’-CCAGCACGATGAAGATCAAG-3’	5’-AGTCCGCCTAGAAGCATTTG-3’	61	171	XM_003124280.5
	STX5	100628048	5’-TGCAGAGTCGTCAGAATGGA-3’	5’-CCAGGATTGTCAGCTTCTCC-3’	61	144	NM_001243381.1
	YWHAZ	780440	5′-TGATGATAAGAAAGGGATTGTGG-3’	5’-GTTCAGCAATGGCTTCATCA-3’	60	203	NM_001315726.1

### Statistical analysis

All statistics were analyzed in R v.4.0.3 [41]. The fetal population was analyzed using a Kruskal–Wallis test to compare the litter sizes between control and treated gilts. Pearson’s Chi-squared Tests were used to compare the frequencies of fetal death and fetal viability between the treated and control groups [42]. The lme4 package fit all linear mixed models (v.1.1.27.1) [43]. Mixed linear regression models were used to test the effect of MMI treatment on ADG, rectal temperatures in gilts, thyroid hormone levels in gilts and fetuses, fetal body weight, individual organ weights, and growth using all live fetuses (*n* = 113) and bone development on select fetuses (*n* = 32). For fetal analysis, group, sex, and fetal preservation were included as fixed effects with litter size as a covariate and gilt as a random effect. Fetal preservation and the interaction between group and sex were removed from the model if not significant. The emmeans package [44] was used to generate pairwise contrasts between groups with Sidak’s correction to account for multiple comparisons when applicable. T4 results were reported in SI units (nmol/L) ± standard deviation. All gene expression data were analyzed non-parametrically with the Kruskal–Wallis test followed by the Wilcoxon rank-sum test for post-hoc comparisons. Expression of three presumptive housekeeping genes (ACTB, STX5, and YWHAZ) was initially evaluated in each tissue, and the two most stable selected based on between group effects and residual correlation. Expression of genes of interest was then normalized to the geometric mean and fold changes calculated using the 2-ΔΔCt method, relative to the mean of the control group. The ggplot2 package [45] was used to visualize all data with significant statistical differences (*P* < 0.05) represented by unique superscripts. Values of *P* > 0.05 and *P* < 0.1 were referred to as trending toward significance.

## Results

### Effects of MMI on maternal physiology

**Figure 2:**
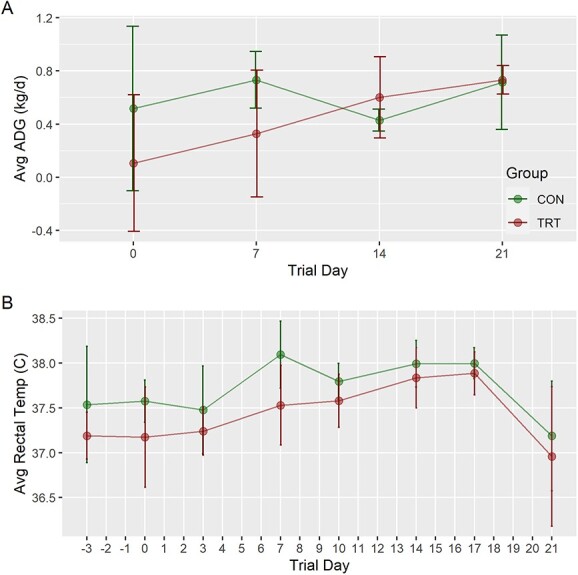
*ADG and average rectal temperature of late gestation pregnant gilts over time.* (A) Mean values of the ADG of late gestation pregnant gilts expressed as kg/day by treatment group (*n* = 4/group). Calculated by body weights measured at trial days −3, 0, 7, 14, and 21. (B) Mean values of rectal temperatures, measured in Celsius, of gilts by treatment group. Measurements were taken biweekly on trial days −3, 0, 3, 7, 10, 14, 17, and 21. Data are presented as line plots with standard error bars. Unique superscripts would signify statistical differences (*P* < 0.05) between groups at any a time point. Absence of superscripts denotes no significant difference was detected between groups at the designated time point.

**Figure 3:**
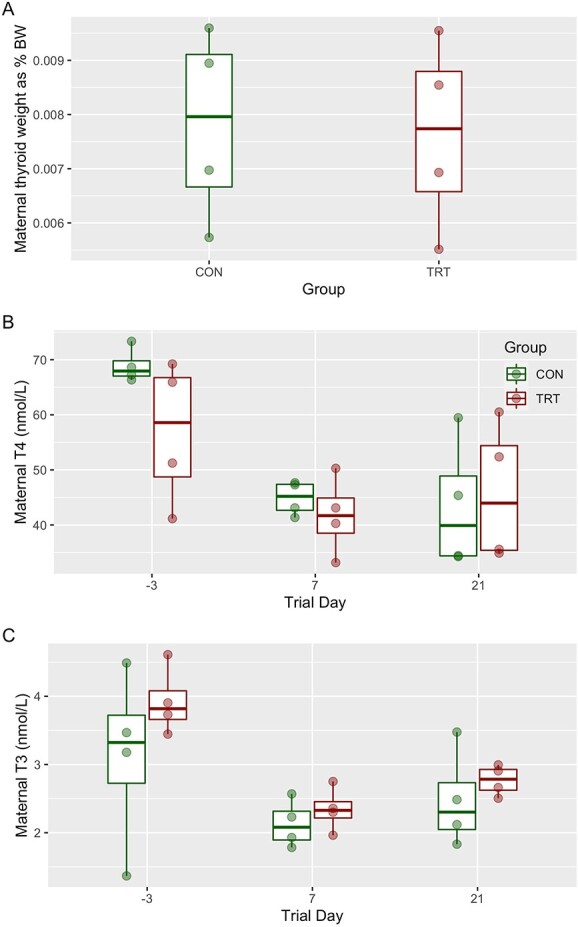
*Relative thyroid weights and circulating thyroid hormone levels over time of late gestation pregnant gilts.* (A) Maternal thyroid weights expressed as a percentage of body weight measured on trial day 21. Individual data points are expressed as boxplots by treatment group (*n* = 4/group). Circulating (B) total T4 and (C) total T3 levels in pregnant gilts were measured via ELISA on trial day −3, 7, and 21. Measurements are expressed in SI units (nmol/L). Data are presented as boxplots groups by treatment (*n* = 4/group) with individual data points. Unique superscripts signify statistical differences between groups at the designated time point (*P* < 0.05). Absence of superscripts denotes no significant difference was detected between groups at that time point.

To determine the effects of MMI on maternal physiology, we evaluated growth, internal temperature, and thyroid status. Over 28 days, ADG for treated gilts ranged from 0.11 (±0.5) to 0.73 (±0.11) kg per day with a mean of 0.44 (±0.43) kg per day, while ADG for control gilts ranged from 0.43 (±0.08) to 0.73 (±0.21) kg per day with a mean of 0.60 (±0.36) kg per day (Figure 2A). No significant difference in ADG was detected between groups at any time. Mean rectal temperatures were trending toward significance between groups (*P* = 0.074), with the average rectal temperature of the treated group being consistently below the average of the control group throughout the trial (Figure 2B). However, post-hoc comparisons revealed no significant differences between groups at any time. No significant difference in thyroid weights relative to body weight was detected between groups (Figure 3A). At trial day –3, before MMI exposure, mean total T3 and T4 levels in maternal serum were 3.92 ± 0.49 and 56.9 ± 13.1 nmol/L, respectively, for the treated group (Figure 3B). Following treatment, T4 levels were significantly depressed at trial day 7 (41.7 ± 7.08 nmol/L, *P* = 0.002) and 21 (45.8 ± 12.7 nmol/L, *P* = 0.004) relative to trial day −3. T4 levels of the treated group were not significantly different between trial days 7 and 21. Total T3 showed a similar pattern, significantly decreasing to 2.34 ± 0.32 (*P* = 0.009) by day 7 and remaining low until day 21. The control gilts showed similar levels of total T3 (3.12 ± 1.29 nmol/L) and T4 (68.9 ± 3.11 nmol/L) as the treated group at trial day −3. Total T4 in the CON groups significantly decreased by trial day 7 (44.9 ± 3.11 nmol/L) and 21 (43.4 ± 11.9 nmol/L). Total T3 in the control group likewise decrease to 2.13 ± 0.35 nmol/L and 2.47 ± 0.72 nmol/L on day 7 and 21, respectively. Most critically, the control group did not significantly differ from the treated group for T3 or T4 at either day 7 or 21.

### Study population

The average litter size did not differ between CON (*n* = 4, 14.8 ± 2.06) and TRT gilts (*n* = 4, 15.5 ± 1.54). The proportion of live (VIA, MEC) to dead (MUM, AUT) fetuses was not significantly affected by treatment. The proportion of viable (VIA) to non-viable (MEC, MUM, AUT) fetuses was not significantly affected by treatment.

### Effects of MMI on the fetal thyroid gland

**Figure 4:**
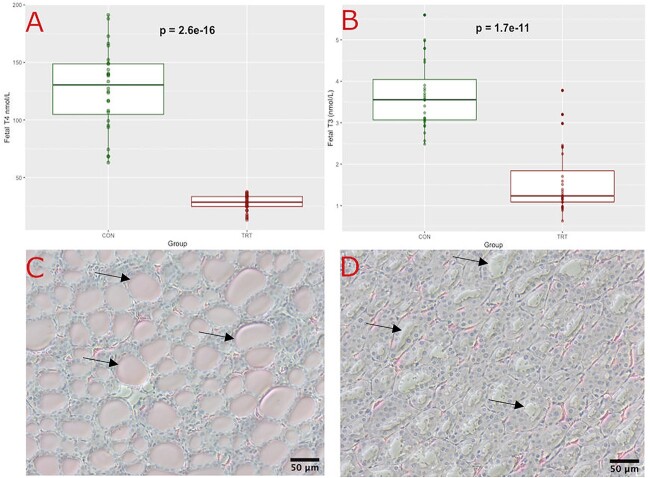
Thyroid hormone levels and thyroid histology from fetuses at trial day 21. Fetal (A) total T4 and (B) total T3 was measured via ELISA using fetal serum collected on trial day 21 and expressed in SI unit (nmol/L). Data are presented as individual data points as a boxplot of control (CON n = 28) and treated (TRT n = 28) groups. Statistical differences between groups is denoted by *P* < 0.05. Hematoxylin and eosin staining of the thyroid gland in fetuses from (C) control and (D) treated gilts, both imaged using a 20× objective (scale bar = 50 μm), arrows indicate a selection of thyroid follicles with (C) and without (D) eosinophilic colloid.

Circulating T4 (*P* < 0.001) and T3 (*P* < 0.001) levels were significantly decreased in fetuses of treated gilts relative to those from control gilts (Figure 4A and B). Analysis by linear mixed model showed no evidence of sex or litter size effect. Visual differences in thyroid histology were evident between fetuses from control (Figure 4C) and treated gilts (Figure 4D). The fetus from a control gilt showed normal histology with large thyroid follicles filled with eosinophilic colloid surrounded by distinct and organized cuboidal follicular cells. In contrast, the fetuses from treated gilt had abnormal thyroid follicles with little to no colloid visible and disorganized follicular cells, consistent with previous with MMI-induced histopathology in rats [31].

### Effects of MMI on fetal growth and development

Fetal body weight was significantly higher in the treated group relative to the control (*P* = 0.015) (Table 2). In terms of fetal organs, the thyroid gland (*P* < 0.001), heart (*P* = 0.024), LVR (*P* = 0.011), KIDs (*P* < 0.001), and spleen (*P* = 0.018) were all significantly heavier in the treated group relative to control (Table 2). The thymus, lungs, and brain weights were not significantly different between groups. Weights of the thyroid glands (*P* < 0.001) and KIDs (*P* < 0.001) were significantly increased when expressed as a percentage of body weight while the relative weight of the heart was significantly decreased (*P* = 0.010). CRL was not significantly different between groups, but girth was significantly larger in the fetuses from treated gilts than those from control (*P* = 0.035) (Table 3). Long bone lengths (including scapula length and width) were measured in the forelimb and hindlimb of select subset fetuses (*n* = 16/group). In the forelimbs, the median values were larger in the fetuses from treated gilts relative to control (Table 3). However, there were no significant differences in the scapula width, length of the scapula, humerus, radius, or ulna. Similarly, lengths of bones in the hindlimbs showed that the median values were consistently smaller in the fetuses from treated gilts relative to control. However, no significant differences were found in the length of the femur, tibia, or fibula.

**Table 2:** Estimated marginal mean (SE) for absolute fetal organ weights and as percentage of body weight by treatment group.

	*Estimated marginal mean (SE)*						
			Weight (g)		Relative to body weight (%)		
		*Estimated marginal mean (SE)*			*Estimated marginal mean (SE)*		
	*n* = 113	CON	TRT	*P*	CON	TRT	*P*
*Fetal body and organ*	Body	880 (28.9)	1029 (28.2)	0.015^*^	–	–	–
	Thyroid	0.18 (0.04)	0.83 (0.04)	<0.001^*^^*^^*^	0.021 (0.006)	0.084 (0.006)	<0.001^*^^*^^*^
	Thymus	1.77 (0.16)	2.25 (0.16)	0.091	0.199 (0.016)	0.217 (0.015)	0.465
	Heart	7.11 (0.21)	8.06 (0.21)	0.024^*^	0.814 (0.007)	0.787 (0.007)	0.010^*^
	Liver	21.7 (0.90)	26.8 (0.89)	0.011^*^	2.45 (0.083)	2.61 (0.081)	0.229
	Lungs	25.0 (1.5)	29.4 (1.47)	0.099	2.83 (0.123)	2.86 (0.120)	0.908
	KIDs	7.35 (0.31)	10.5 (0.31)	<0.001^*^^*^^*^	0.839 (0.013)	1.025 (0.013)	<0.001^*^^*^^*^
	Spleen	1.1 (0.09)	1.52 (0.08)	0.018^*^	0.127 (0.007)	0.147 (0.007)	0.095
	Brain	29.5 (0.58)	30.5 (0.57)	0.289	3.53 (0.135)	3.07 (0.132)	0.064

CON, from control gilt; TRT, from treated gilt. ^*^ Significance of *P* < 0.05, ^*^^*^ Significance of 0.001 < P < 0.01, ^*^^*^^*^ Significance of *P* < 0.001.

**Table 3:** Estimated marginal mean (SE) for fetal phenotypes by treatment group.

		*Estimated marginal mean (SE)*		
		CON	TRT	*P*
*Fetal Growth (mm)*	Crown rump length	345 (3.21)	354 (3.14)	0.089
	Girth	78.7 (1.18)	83.5 (1.16)	0.035^*^
*Fetal Bone Length (cm)* $^{\textrm{T} \!\!\!\!- }$	Scapula (length)	2.96 (0.08)	3.13 (0.08)	0.193
	Scapula (width)	2.06 (0.08)	2.27 (0.07)	0.122
	Humerus	2.97 (0.07)	3.05 (0.07)	0.452
	Radius	2.03 (0.05)	2.10 (0.05)	0.422
	Ulna	2.66 (0.06)	2.71 (0.06)	0.615
	Femur	5.42 (0.35)	4.67 (0.35)	0.141
	Tibia	5.23 (0.35)	4.49 (0.35)	0.15
	Fibula	4.57 (0.30)	3.92 (0.30)	0.143

CON, from control gilt; TRT, from treated gilt. $^{\textrm{T} \!\!\!\!-}$ n = 32. ^*^Significance of *P* < 0.05.

Sex had a significant effect on fetal phenotypes, with maleness associated with an increase in body (*P* = 0.02), LVR (*P* = 0.001), lung (*P* = 0.01), spleen (*P* = 0.05), and brain (*P* = 0.05) weights (Table 4). Male fetuses also had significantly larger CRL (*P* < 0.001) and girth (*P* = 0.004) than female fetuses. Sex did not significantly affect the length and width of the scapula or the length of long bones in neither forelimb nor hindlimb. Circulating levels of both T3 and T4 were not affected by sex of the fetus. All mixed models were averaged over sex because of significant associations with fetal body weight and size.

**Table 4:** Parameter estimates for the effect of MMI treatment (TRT), Sex (Male), and litter size on fetal organ weights, growth, bone, and thyroid hormone measurements.

	*Predictors*						
		Group (Effect of TRT)		Sex (effect of Male)		Litter Size (effect of each additional fetus)	
		Estimate	*P*	Estimate	*P*	Estimate	*P*
*Fetal Body and Organ Weights (g)*	Body	149.15	0.015^*^	82.28	0.024^*^	−62.2	0.001^*^^*^
	Thyroid	0.66	<0.001^*^^*^	0.04	0.095	−0.01	0.403
	Thymus	0.48	0.091	0.05	0.734	−0.22	0.013^*^
	Heart	0.95	0.024^*^	0.5	0.063	−0.55	<0.001^*^^*^^*^
	Liver	5.14	0.011^*^	3.09	0.001^*^^*^	−1.9	0.002^*^^*^
	Lungs	4.3	0.099	2.92	0.011^*^	−1.65	0.029^*^
	KIDs	3.15	<0.001^*^^*^^*^	0.45	0.222	−0.48	0.007^*^^*^
	Spleen	0.42	0.018^*^	0.13	0.047^*^	−0.07	0.062
	Brain	0.98	0.289	0.74	0.048^*^	−0.18	0.439
*Fetal Growth (mm)*	Crown rump length	9.59	0.089	13.85	<0.001^*^^*^^*^	−6.57	0.002^*^^*^
	Girth	4.8	0.035^*^	2.6	0.004^*^^*^	−2.27	0.003^*^^*^
*Fetal Bone Length (cm)*	Scapula (length)	0.18	0.193	0.02	0.814	−0.05	0.159
	Scapula (width)	0.21	0.122	−0.01	0.913	−0.03	0.311
	Humerus	0.08	0.452	0.03	0.702	−0.04	0.176
	Radius	0.06	0.422	0.02	0.719	−0.02	0.348
	Ulna	0.05	0.615	0.03	0.680	−0.03	0.226
	Femur	−0.76	0.141	0.09	0.854	0.07	0.610
	Tibia	−0.74	0.150	0.1	0.834	0.06	0.670
	Fibula	−0.65	0.143	0.1	0.822	0.07	0.547
*Fetal Thyroid Hormones (nmol/L)*	T4	−96.80	<0.001^*^^*^^*^	−0.006	0.999	−2.22	0.052
	T3	−2.13	<0.001^*^^*^^*^	−0.07	0.758	0.033	0.671

^*^ Significance of *P* < 0.05), ^*^^*^ Significance of 0.001 < *P* < 0.01, ^*^^*^^*^ Significant of *P* < 0.001.

Litter size was consistently associated with reduced fetal body weight, with each incremental increase in litter size associated with an estimated reduction of 62.2 g. This effect was largely consistent across phenotypic effect including fetal length, organ weight, and bone length, with (Table 4). The fetal thyroid and brain were the only two organs not significant or trending toward significance with all other organs showing significant decreased weights with increasing litter size. Fetal bone length in the hindlimb was consistently positively influenced by increasing litter size, although not a significant effect.

Fetal preservation of live fetuses (VIA and MEC) had no significant effect on fetal body or organ weights, fetal body size, or fetal bone measurements and was removed as a fixed effect from all models during analysis (Table 4).

### Effects of MMI on fetal thyroid hormone metabolism gene expression

To assess the effects of treating pregnant gilts with MMI on fetal thyroid hormone metabolism, we used qPCR to evaluate gene expression of three deiodinases and two known transporters of thyroid hormone in a subset of live fetuses (*n* = 32).

**Figure 5:**
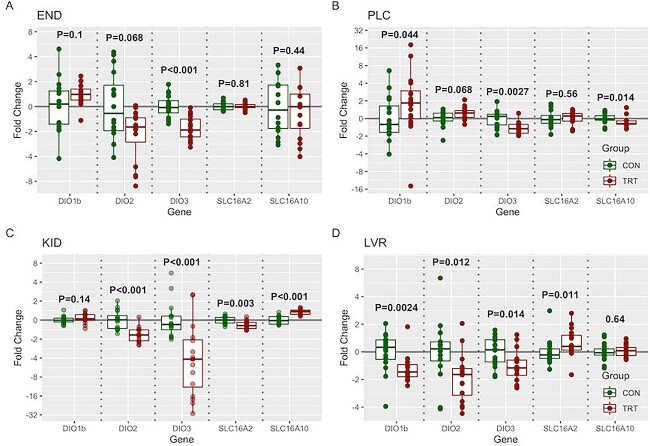
*Fetal gene expression of three deiodinase and two transport genes associated with thyroid hormone.* Gene expression was measured in tissues including (A) END, (B) fetal portion of the PLC, (C) fetal KID, and (D) fetal LVR. Fetuses (*n* = 16/group) were selected based on viable status and central location in the uterine horn. Fold changes were calculated within tissue relative to the average of the control group. Significant statistical differences recognized at *P* < 0.05.

The END corresponding to fetuses from treated gilts had a significant downregulation of the deactivating DIO3 relative to those from control gilts ($\overset{\sim }{x}=-$1.95-fold, *P* < 0.001) (Figure 5A). Expression of DIO1 (*P* = 0.1) and DIO2 (*P* = 0.068) in the fetuses from treated gilts was approaching significant upregulation and downregulation, respectively. The expression of transporters SLC16A2 and SLC16A10 was not significantly different in fetuses from treated gilts relative to control.

The fetal portion of the placenta had significant downregulations in expression of DIO3 ($\overset{\sim }{x}=-$1.05-fold, *P* = 0.003) and SLC16A10 ($\overset{\sim }{x}=-$1.02-fold, *P* = 0.014) in fetuses from treated gilts relative to control (Figure 5B). Expression of DIO1 was significantly upregulated in fetuses from treated gilts relative to control ($\overset{\sim }{x}=1.85$-fold, *P* = 0.044). Expression of DIO2 was approaching significant upregulation (*P* = 0.068). The expression of transporter SLC16A2 was not significantly different in fetuses from treated gilts relative to control.

The KID of fetuses from treated gilts saw significant changes in expression relative to fetuses from control gilts for every gene evaluated except DIO1 (Figure 5C). There was a significant upregulation in the expression of SLC16A10 ($\overset{\sim }{x}=$1.0-fold, *P* < 0.001). Significant downregulations were seen in the expression of DIO2 ($\overset{\sim }{x}=-$1.5-fold, *P* < 0.001), DIO3 ($\overset{\sim }{x}=-$5.05-fold, *P* < 0.001), and SLC16A2 ($\overset{\sim }{x}=-$0.82-fold, *P* = 0.003).

The fetal LVR showed significant downregulations of DIO1 ($\overset{\sim }{x}=-$1.5-fold, *P* = 0.002), DIO2 ($\overset{\sim }{x}=-$1.6-fold, *P* = 0.012), and DIO3 ($\overset{\sim }{x}=-$1.1-fold, *P* = 0.014) expression in the fetuses from treated gilts relative to control (Figure 5D). Expression of SLC16A2 ($\overset{\sim }{x}=$0.4-fold, *P* = 0.011) was significantly upregulated in the fetuses from treated gilts relative to control while no significant difference was seen in the expression of SLC16A10.

## Discussion

The hemochorial type of placenta found in humans and rodents is readily permeable to macromolecules such as fatty acids and immunoglobulins due to the direct contact of maternal blood with the fetal chorion [7]. It has been well documented that this invasive placenta type is also permeable to MMI, which can be used experimentally to produce congenital hypothyroidism in the developing conceptus [32, 33, 35]. In contrast, the epitheliochorial type of placenta found in pigs has intact maternal and fetal tissue layers, with additional barriers separating maternal and fetal blood supplies. This separation prevents the passage of immunoglobulins and results in a low exchange of macromolecules, like fatty acids [7]. However, to the authors’ knowledge, the current study is the first to investigate the permeability of a non-invasive epitheliochorial placenta to MMI by observing the effects on the fetal thyroid status. We observed mean values of circulating fetal thyroid hormones were decreased by 75.3% in T4 and by 56.9% in T3 relative to fetuses that were not exposed to MMI, which is consistent with congenital hypothyroidism in humans [46]. Within the thyroid of fetuses from treated gilts, we observed misshapen follicular cells surrounding follicles containing a lack of colloid. These findings are consistent with histological signs of goiter as seen in both hypothyroid humans [47] and rats exposed to MMI [31]. Collectively, we can conclude that the current study shows, for the first time, that MMI crosses the epitheliochorial placenta of the pig fetus and renders it hypothyroid.

Hypothyroidism in adult animals is known to manifest as weight gain, cold intolerance, low thyroid hormone levels, and, in some cases, a goitrous thyroid gland [48, 49]. Although the current study has established fetal hypothyroid status after dam MMI exposure, we saw few effects on maternal physiology. No significant differences in ADG or thyroid size were detected between groups. Although also not significant, we did observe that the average rectal temperatures were consistently lower in treated gilts than in control, which is consistent with cold intolerance seen in hypothyroid humans [48]. Maternal T4 and T3 levels in serum did not significantly differ between groups at any point measured in the current study. However, T4 and T3 levels in both groups dropped in tandem from trial day −3 to 7 and remained stable through trial day 21. Temporal changes have been previously seen in the circulating T4 of healthy post-natal pigs [26] and in maternal T4 during early pregnancy in humans [34]. Interestingly, we have previously observe a similar, although less acute, decrease in circulating T4 in pregnant gilts during the same period of gestation [25]. As the present trial was not conducted in a climate controlled facility, the exacerbated effect may be due environmental factors such as ambient temperature, which has been associated with changes in thyroid hormone levels in healthy post-natal pigs [50]. It is worth noting that our animals were housed side by side throughout the trial, so that any environmental changes would equally impact the entire experimental population. A combination of environmental and gestational effects are most likely the cause of the observed drop in T4 of both control and treated animals. In contrast to the rodent models of congenital hypothyroidism using MMI, the lack of significant maternal effects in the present model suggests that the pregnant gilt has the resources to compensate the hypothyroid fetuses.

Many clinical reports suggest that MMI has rare teratogenic effects on human fetuses in early pregnancy, such as esophageal atresia, eye deformities, and delayed neurodevelopment [51, 52]. In humans, it is recommended that those experiencing Graves’ Disease during pregnancy take PTU during the first trimester and switch to MMI in the second trimester for the remainder of the pregnancy. This is thought to avoid suspected embryopathy associated with MMI exposure during early gestation and minimize any hepatotoxic effects associated with PTU [53–55]. However, the literature is limited concerning the possible teratogenic effects of MMI or other anti-thyroid compounds, such as PTU, on developing fetuses in late gestation. In adults, cytotoxicity is standard during hypothyroidism and appears to be enhanced if induced by MMI in rats [29], suggesting that MMI has a cytotoxic and tissue-damaging effect. Despite the intensive and detailed necropsy procedure utilized in the current study, we found no evidence of congenital deformities or fetal compromise following MMI exposure during days 85–106 of gestation in swine. While the secondary effects of MMI on the fetus remain uncertain and require more targeted analysis, the results of the present study demonstrate its utility as a model for generating non-pathogenic hypothyroidism in the late gestation fetus.

In the current study, we observed that the absolute and relative weight of KIDs from fetuses of MMI-treated gilts were significantly increased. This is consistent with increased KID weights in thyroidectomized fetal sheep [56]. Interestingly, MMI in adult rats has also shown signs of cellular damage and edema of the KIDs [29, 57]. This suggests that the enlarged KIDs in the current study could be an effect of hypothyroidism further exacerbated by exposure to MMI, possibly due to increased renal activity associated with drug excretion. While MMI-induced hypothyroidism has also been associated with hepatotoxicity in humans [58, 59], we saw no apparent change in the organs in the present study. This suggests that MMI is not hepatotoxic in fetal pigs but could be nephrotoxic.

Fetuses with low placental permeability, such as sheep, exposed to hypothyroidism in utero commonly have decreased body weight relative to euthyroid fetuses [16]. In contrast, placentas with high permeability, such as in humans and rats, allow for a greater exchange of maternal thyroid hormone to supplement the fetus during hypothyroidism. As a result, the body weights of rat fetuses with MMI-induced congenital hypothyroidism are often normal [35]. A similar effect is seen in naturally occurring congenital hypothyroidism detected at birth in humans [16]. Interestingly, the current study observed significant increases in body weight and girth, but not CRL, of hypothyroid pig fetuses in utero. This is consistent with increased body weight seen in adult pigs with MMI-induced hypothyroidism [30], and increased weight is a common clinical sign of hypothyroidism in adult humans [60]. In addition, our findings suggest that hypothyroid fetuses experience non-allometric growth. While not measured in the present study, this may indicate an increase in fetal fat deposition in response to hypothyroidism. Such a response would be consistent observations following ovine fetal thyroidectomy, which results in an increased deposition of perirenal adipose tissue [61].

Thyroidectomy of late gestation fetal sheep showed a decrease in the proportion of fetal heart weight relative to body weight, which is associated with decreased cardiomyocyte maturation [56]. Similarly, in fetal pigs experiencing a decrease in circulating thyroid hormone associated with PRRSV infection, the heart is severely impacted by altered gene expression associated with the regulation of cell cycle progression [25, 62]. These reports are consistent with current study findings that fetuses rendered hypothyroid from MMI exposure have decreased heart weights relative to body weight. While the full extent of the influence MMI has on the fetal pig heart requires a more direct investigation, this study shows that the hearts of fetuses exposed to MMI do not grow proportionally with body weight.

Thyroid hormone is essential for proper fetal bone development during gestation in many mammalian species and has been extensively studied in humans and rodents [63, 64]. As a result, short stature is a common consequence of congenital hypothyroidism in humans [65]. A similar effect is seen in thyroidectomized fetal sheep with significantly decreased long bone lengths relative to intact fetuses [66]. Very few studies have investigated the effect of hypothyroidism on fetal bone growth in swine. However, one study found decreased ossification in PRRSV infected and meconium-stained fetuses, which were, by extension, in an NTIS like state [39]. Interestingly, the current study shows no significant differences between control and hypothyroid fetuses in any of the measured forelimb or hindlimb long bone lengths. This inconsistency could be due to the early development of the thyroid gland in fetal swine relative to other species, like humans [14, 67]. The early accessibility to thyroid hormone would suggest that fetal bone development is stimulated earlier in gestation than in humans and sheep and therefore is less affected by hypothyroidism during late gestation.

There is a sex-specific effect related to the regulation of thyroid hormone in adult humans in which females show more thyroid hormone dysregulation than males [68, 69]. However, little evidence has been found to support a similar effect in fetuses or at what point the effect is apparent during development. A previous fetal swine investigation reported that females have significantly higher T4 levels than their male counterparts regardless of PRRSV infection or genotype [39]. In contrast, the current study found no evidence of a sex effect on circulating T4 levels measured in 62 female and 50 male fetuses. The hypothesis that male fetuses are more susceptible than females to their environment and infections could explain this inconsistency [70]. Therefore, fetal PRRSV infection could greatly accentuate the sex effect on thyroid hormones, making males more susceptible to viral and chronic effects than females.

During hypothyroidism, gene expression of deiodinases in adult and fetal rats has been noted to change in a compensatory response through increasing activation (DIO2) and decreasing deactivation (DIO3) of thyroid hormone [71, 72]. Similarly, excess T4 has been shown to reduce expression of DIO2, while T3 results in the opposite effect [73]. Regulation of these genes by their substrates is may be the result of thyroid hormone response elements, which have for instance been definitively identified in the promoter region of DIO3 [74]. Such feedback mechanisms allow for local regulation of T3 availability within target tissues which server to offset changes in systemic availability. Deiodinases are the major component of the placental enzymatic barrier to maternal thyroid hormone in swine, which deactivates maternal thyroid hormone and prevents its passage to the fetus [23]. We have recently demonstrated, that in fetal pigs experiencing NTIS in response to PRRSV infection, metabolism of thyroid hormone at the MFI and within fetal organs is upregulated further exacerbating the depressed thyroid hormone state [40]. In contrast, the current study shows more compensatory alterations to thyroid metabolism indicating the fetus is in a truly hypothyroid state.

## Conclusions

The present study clearly demonstrates that maternal treatment with MMI produces profound fetal hypothyroidism in the late gestation pig with limited impact on the dams thyroid status. We observed significantly increased fetal thyroid gland weights, with histological evidence of goiter, and significant decrease in circulating T4 and T3 levels, all indicative of hypothyroidism in the fetus. In addition, the hypothyroid fetuses experienced non-allometric growth as evidenced by a significant increase in body weight and girth without a corresponding increase in crown rump or bone length. Additionally, the significant decrease in DIO3 expression in all tissues evaluated indicates a compensatory response, increasing access to maternal thyroid hormone and decreasing fetal metabolism of the hormone. Future investigations conducting more targeted analysis of the fetal KID and heart may be valuable in determining the full impact of MMI-induced hypothyroidism on these vital organs’ growth rate and development.

## Data Availability

The datasets generated during and/or analyzed during the current study are available from the corresponding author on reasonable request.
